# Sex-related differences in phenotype and nigro-striatal degeneration of c-rel^-/-^ mouse model of Parkinson’s disease

**DOI:** 10.1186/s13293-025-00761-0

**Published:** 2025-10-10

**Authors:** Edoardo Parrella, Vanessa Porrini, Michele Mario Gennari, Marina Benarese, Federico Del Gallo, Anna Andrioli, Chiara Fritzsch, Marina Bentivoglio, Paolo Francesco Fabene, Marina Pizzi

**Affiliations:** 1 Division of Pharmacology, Department of Molecular and Translational Medicine, University of Brescia, Brescia, Italy; 2 Departmental Faculty of Medicine, Saint Camillus International University of Health Sciences, Rome, Italy; 3 Section of Innovation Biomedicine, Department of Engineering for Innovation Medicine, University of Verona, Verona, Italy; 4 Section of Anatomy and Histology, Department of Neurosciences, Biomedicine and Movement Sciences, School of Medicine, University of Verona, Verona, Italy

**Keywords:** Parkinson’s disease, Sexual dimorphism, NF-κB/c-Rel, Nigrostriatal degeneration, DAT, Motor symptoms, Non-motor symptoms

## Abstract

**Background:**

Sex is an important factor in the development and symptom expression of Parkinson’s disease (PD). Risk of developing PD, motor and non-motor symptoms and response to treatment differ between men and women, with women showing lower disease incidence, later onset of motor deficits and generally milder symptoms than men. We previously reported that male mice lacking the NF-κB/c-Rel protein (c-rel^-/-^ mice) undergo age-related accumulation of α-synuclein, and loss of dopaminergic neurons, in the substantia nigra (SN). In addition, c-rel^-/-^ male mice present a progressive PD-like phenotype characterized by both motor deficits and non-motor symptoms (such as constipation, hyposmia, anxiety, depressive-like behavior and apathy). In this study, we give evidence that female mice reproduce only part of the parkinsonian pathology and do not show behavioral manifestations.

**Methods:**

Nigro-striatal alterations as well as motor and non-motor symptoms were assessed in aged c-rel^-/-^ and wild-type (wt) male and female mice through histological techniques and behavioral tests.

**Results:**

Likewise c-rel^-/-^ males, c-rel^-/-^ females displayed significant reduction of dopaminergic neurons in the SN at 18 months of age, but only minor reduction of striatal TH-positive (TH+) and DAT-positive (DAT+) dopaminergic fibers compared to wt littermates. Besides, c-rel^-/-^ females did not develop significant motor deficits and non-motor symptoms, as constipation, hyposmia, depressive-like and apathetic behaviors.

**Conclusions:**

Our results show that, differently from aged males, c-rel^-/-^ females do not develop a parkinsonian behavior, in line with evidence from the human PD. The phenotype mice display a nigral dopaminergic neuron degeneration but conserved nigrostriatal fiber density. The degeneration and PD-like symptoms are compatible with the sex-related differences on incidence and symptoms progression observed in PD patients.

**Supplementary Information:**

The online version contains supplementary material available at 10.1186/s13293-025-00761-0.

## Abstract (plain language summary)

In many neurodegenerative diseases there are sex differences in terms of incidence, onset and severity of symptoms. This is true also for PD, a pathology in which women display lower disease incidence, later onset of motor deficits, and generally milder symptoms than men. We previously demonstrated that male c-rel^-/-^ mice exhibit progressive parkinsonian features associated with the loss of neurons in the nigrostriatal pathway, alongside the emergence of motor dysfunctions and non-motor disturbances, such as hyposmia, constipation and neuropsychiatric impairments. Here, we examined if also c-rel^-/-^ female mice reproduce the pathology and the symptomatology observed in males. We discovered that aged c-rel^-/-^ female mice showed loss of dopaminergic neurons in the SNpc, but without a marked reduction of striatal dopaminergic fibers. Additionally, c-rel^-/-^ female mice displayed neither significant motor defects, nor significant non-motor symptoms including constipation and hyposmia. Finally, the neuropsychiatric deficits were also milder in c-rel^-/-^ females. Overall, our results indicate a sexual dimorphism in the c-rel^-/-^ mouse model of PD, with females showing milder pathology and symptoms as observed in PD patients.

**Supplementary Information:**

The online version contains supplementary material available at 10.1186/s13293-025-00761-0.

## Highlights

- Similarly to c-rel^-/-^ male mice, aged c-rel^-/-^ females exhibit degeneration of dopaminergic neurons in the SN. However, they do not display significant reduction of striatal dopaminergic fibers, as observed in c-rel^-/-^ males.
- Aged c-rel^-/-^ females do not show significant motor deficits when compared to c-rel^-/-^ males.
- In contrast to c-rel^-/-^ males, aged c-rel^-/-^ females do not develop notable non-motor deficits, such as constipation and hyposmia, and experience less severe neuropsychiatric manifestations.

**Supplementary Information:**

The online version contains supplementary material available at 10.1186/s13293-025-00761-0.

## Background

With an estimated prevalence of 1–2 cases per 1000 people, Parkinson’s disease (PD) is the most widespread movement disorder and it is primarily associated with motor dysfunction, including bradykinesia, resting tremor, and rigidity [1]. In addition to motor symptoms, PD is also associated with numerous non-motor manifestations, including constipation, hyposmia, sleep disturbances, anxiety, and depression, which can precede motor deficits by several years [2]. The defining pathological features of PD include the gradual loss of dopaminergic neurons in the substantia nigra pars compacta (SNpc) and the aggregation of α-synuclein within Lewy bodies [3].

As observed in other neurodegenerative conditions, PD exhibits notable sex-related differences in prevalence, onset, and clinical presentation [4–8]. Epidemiological studies indicate that men are more likely than women to develop PD [9–11]. Furthermore, the disease typically progresses more rapidly in males, with earlier onset of motor symptoms [10]. Men with PD tend to exhibit a more severe postural instability-dominant phenotype, which includes gait disturbances, freezing of gait, and an increased tendency to fall [10, 12]. Additionally, male patients show a heightened risk of developing camptocormia, a postural abnormality characterized by forward trunk flexion when standing or walking [13]. In contrast, female patients more frequently present with a tremor-dominant phenotype, which is often associated with a milder disease course [10, 12, 14]. However, women display a heightened sensitivity to levodopa therapy, leading to an increased incidence of levodopa-induced dyskinesias [15–17]. Sex-based differences are also evident in the early non-motor manifestations of drug-naïve PD patients, with men exhibiting more pronounced olfactory and cognitive impairments, sleep disturbances, and severe drooling [12, 18–25]. Conversely, women are more likely to experience dysphagia [26], as well as persistent and severe anxiety and depression [22, 27–30].

The regulation of a broad spectrum of physiological and pathological processes is governed by the nuclear factor kappa-light-chain enhancer of activated B cells (NF-κB) transcription factor [31, 32]. This protein family comprises five distinct subunits - RelA, RelB, c-Rel, NF-κB1 and NF-κB2 - that form transcriptionally active dimers [33, 34]. The functional outcome of NF-κB activation, whether pro- or anti-apoptotic, depends on the specific dimer composition [35–37]. An aberrant modified form of RelA, acRelA(K310), which retains acetylation at lysine 310 while undergoing deacetylation at other lysine residues, has been implicated in the induction of pro-apoptotic genes in preclinical models of brain ischemia [38, 39]. Conversely, the inclusion of c-Rel within NF-κB dimers has been associated with neuroprotection in experimental models of brain ischemia [40, 41] and neurotoxicity [42–44].

Recent findings suggest that an imbalance between NF-κB subunits may contribute to PD pathology. Studies have shown that post-mortem SN samples from PD patients exhibit increased Ac-RelA(K310) levels, alongside reduced DNA-binding activity of c-Rel in both the SN and peripheral blood mononuclear cells (PBMCs) [45]. Supporting this hypothesis, c-Rel-deficient (c-rel^-/-^) male mice display elevated Ac-RelA(K310) in the striatum [35] and develop an age-dependent pathology that mirrors key PD features [46–49]. Alpha-synuclein deposition in these mice first emerges in the enteric nervous system (ENS) and olfactory bulb (OB) at 2 months [48], subsequently spreads to the dorsal motor nucleus of the vagus (DMV) and locus coeruleus (LC) at 5 months [48], and is later detected in the SNpc by 12 months [46]. A decline in striatal dopamine transporter (DAT) levels is also evident at this stage [48]. By 18 months, these mice exhibit a significant reduction in SNpc dopaminergic neurons and a concomitant loss of striatal dopaminergic fibers [46]. Additionally, a mild neuroinflammatory response precedes nigral degeneration [47], transitioning into more pronounced microglial engagement in the SNpc and striatum at 18 months [46].

In young male c-rel^-/-^ mice, early-stage α-synuclein accumulation in the ENS and OB is linked to the development of prodromal features such as hyposmia and constipation [48]. By 12 months, these mice also develop anxiety- and depression-like behaviors, followed by apathy at 18 months [49]. At this later stage, motor deficits emerge and respond to levodopa treatment [46].

While previous research has yielded critical insights into the contribution of c-Rel to PD pathogenesis, these studies have exclusively focused on male mice. Consequently, it remains unclear whether female c-rel^-/-^ mice develop similar PD-related pathology and symptoms. To address this gap, the current study assesses nigrostriatal degeneration and its associated behavioral impairments, including gait abnormalities, constipation, hyposmia, and neuropsychiatric features in male and female c-rel^-/-^ mice between 18 and 23 months of age, a time window where PD-like pathology and symptoms are well documented in this model [46–49].

## Materials and methods

### Animals

C57BL/6 mice carrying a null mutation for the c-Rel gene (c-rel^-/-^ mice) were previously generated and characterized [46, 50]. Animals were maintained in groups of 2–4 per cage under standard housing conditions, including a 12-hour light/dark cycle, 55% humidity, and an ambient temperature of 22–23 °C. Each cage was supplied with bedding materials and red mouse shelters (Tecniplast). Animals were provided with a standard laboratory diet (complete feed for mice and rats 4RF21, Mucedola) and water *ad libitum*. All experimental procedures received approval from the Animal Research Committees of the University of Brescia and were conducted following the EU regulations on animal research (2010/63/EU). Ethical guidelines of the University of Brescia were strictly followed.

The animals used in this study were tested at an age greater than 18 months. Since in female mice the transition to reproductive senescence typically occurs between 9 and 12 months of age, the female mice analyzed in this study had ceased their estrous cycles and were considered reproductively senescent [51–54].

### Immunohistochemistry

For histological analyses, chloral hydrate was used to anesthetize mice, which were subsequently perfused transcardially with phosphate-buffered saline (PBS). Fixation was performed with 4% paraformaldehyde. Extracted brains were subjected to cryoprotection in 30% sucrose, then sectioned coronally at 40 μm thickness for the SN and 30 μm for the striatum using a freezing microtome at specific stereotaxic coordinates as previously described [55].

One series of SN sections was processed for immunohistochemical detection of tyrosine hydroxylase (TH). Endogenous peroxidase activity was blocked by treating brain sections with 1% H_2_O_2_ for 15 min. After 1 h incubation with 5% normal goat serum (NGS), sections were incubated overnight at 4 °C with anti-TH antibody (sc-14007, Santa Cruz Biotechnology, 1:500) in 1% NGS and 0.3% Triton-X-100 in PBS.

After washing, sections were incubated with biotinylated goat anti-rabbit IgG (Vector Labs, 1:200) for 2 hours. To visualize the immunosignal, avidin-biotin-peroxidase complex (Vectastain, Vector Labs) and 3-3’-diaminobenzidine (DAB, Sigma-Aldrich) as a chromogen were used. Blind-coded slides were used for unbiased analysis. Stereological counting of TH+ neurons in the SN was performed using the Stereo Investigator system (MBF Bioscience). Regions of interest were outlined at 10x magnification under an Olympus BX53 microscope equipped with a Retiga 2000R CCD camera. Systematic random sampling was applied using a 100/100 µm grid. Cells within the counting frame (70/70 µm width/height; 10 μm depth) were quantified, excluding those touching specific frame borders. To estimate the total population of TH+ neurons within the SN, the template described by Ip et al. was applied to the cell count data [56].

One series of SN sections was processed for Nissl staining. Sections were incubated with 0.5% cresyl violet (Sigma-Aldrich) solution for 1,5 min and subsequentially dehydrated in 90–100% ethanol and xylene. Then, sections were mounted with DPX (Fluka). To determine the number Nissl-stained cells in the SN, sections were observed in bright field microscopy. All values were normalized to the mean of wt males and expressed as relative changes.

For immunofluorescence, TH-DAPI or dopamine transporter (DAT)-DAPI staining were evaluated in two series of striatal sections as previously described [57]. Sections were permeabilized with 20% methanol and 0.3% Triton X-100 in PBS, blocked for 1 h in 2% NGS, 3% BSA, 0.3% Triton X-100 in PBS, and incubated overnight at 4 °C with primary antibodies [TH (sc-14007) or DAT (AB5802, Merck Millipore)]. Then, sections were rinsed and subsequently incubated at room temperature for 1 h with a Cy3-conjugated secondary antibody (1:3000, Jackson ImmunoResearch). Lipofuscin autofluorescence was quenched using TrueBlack (Biotium). Sections were mounted with Vectashield (Vector Laboratories). Analyses were carried out using a Zeiss LSM 510 confocal microscope (Carl Zeiss) and the acquisition parameters during confocal imaging were maintained constant for all the images acquired. A total of five sections per mouse were analyzed, averaging eight fields per section. A fixed threshold was set in FIJI to exclude background signal and applied to each digitized image in order to evaluate the optical density of TH and DAT staining. Data from TH and DAT analysis were expressed as the positive area in each sample (µm^2^).

### Colon length measurement

Cohorts of 18– and 19-month-old wt and c-rel^-/-^ mice were used to determine colon length. Mice euthanasia was performed by cervical dislocation and the colon was recovered. The length from the ileocecal junction to the anal verge was recorded [58].

### Behavioral assessments

Separate cohorts of wt and c-rel^-/-^ male and female mice underwent behavioral evaluations to assess colon motility, hyposmia, anxiety, depressive-like behaviors, apathy, and gait deficits, as previously described [46, 49]. Mice used for colon motility tests were also evaluated for food and water intake. All tests were performed under controlled environmental conditions (22–23 °C, 55% humidity, 12-hour light/dark cycle). Testing occurred during either the light or dark phase as appropriate. Equipment was cleaned with ethanol 70% between trials to prevent olfactory cues. Researchers were blinded to genotype during testing and analysis.

### Catwalk gait analysis

Gait parameters of 20–23-month-old mice were analyzed using the CatWalk 7.1 system (Noldus) [46]. Mice underwent a three-day training period before test day. On test day, three uninterrupted runs were performed to record multiple parameters (run duration, run speed, swing speed, stand duration, and stride length). Data were analyzed using a pixel threshold of ≥ 25 arbitrary units.

### Colon motility, food, and water intake

Colon motility was assessed in 18–19-month-old mice through the one-hour stool collection assay. Stool frequency and water content were measured relative to body weight (bw). Food and water intake were monitored in the same cohort over two additional days by measuring daily consumption, normalized to bw.

### Olfactory function

Hyposmia was evaluated using the odor detection test. Mice were exposed to increasing concentrations of a novel odorant (vanilla extract) across three daily sessions. A video-tracking system (Ugo Basile ANY-Maze) recorded the time spent sniffing odor- and water-containing cartridges. Odor preference and exploratory behavior were quantified as the percentage of time spent sniffing the scented cartridge and total sniffing duration, respectively.

### Anxiety assessment

Anxiety-like behavior was assessed using the open field (OF) test. Mice were placed in a 40 × 40 × 40 cm black plastic arena, and their movements were tracked for 5 min. Time spent in the center, percentage of distance traveled, and peripheral zone exploration (thigmotaxis) were measured. Reduced center exploration was interpreted as increased anxiety.

### Depressive-Like behavior

Depressive-like behavior was analyzed using the Forced Swim Test (FST) with a Water Wheel. Mice were placed in a water tank (25 ± 1 °C) equipped with a freely rotating wheel. Floating behavior, latency to immobility, and total immobility duration were manually scored, while wheel rotations, turning time, and revolution speed were recorded automatically using Ugo Basile ANY-Maze software.

### Nest Building test

Motivational and goal-directed behavior were assessed via the nest-building test. Mice housed in groups (2–4 per cage) were provided with a cellulose bag filled with nesting material. The time taken to gnaw, empty the bag, and construct a nest was recorded at 30-minute intervals by a blinded observer.

### Statistical analysis

GraphPad Prism was used to perform data analysis. Results are presented as mean ± standard error of the mean (SEM), with *p* < 0.05 considered statistically significant. Normal distribution of data was assessed with Shapiro-Wilk test. Two-way ANOVA followed by Sidak’s post-hoc test, or Kruskal-Wallis test with Dunn’s post-hoc test, was used where appropriate. One-sample t-tests were performed in the odor detection test to compare odor sniffing against the 50% chance level. Effect sizes are presented as partial eta squared (η_p_^2^) for ANOVAs, Cohen’s d (*d*) for t-tests and eta squared (η^2^) for Kruskal-Wallis tests.

## Results

### Aged c-rel^-/-^ males and females display similar degeneration of dopaminergic neurons in the SN but different loss of striatal dopaminergic fibers

**Fig. 1:**
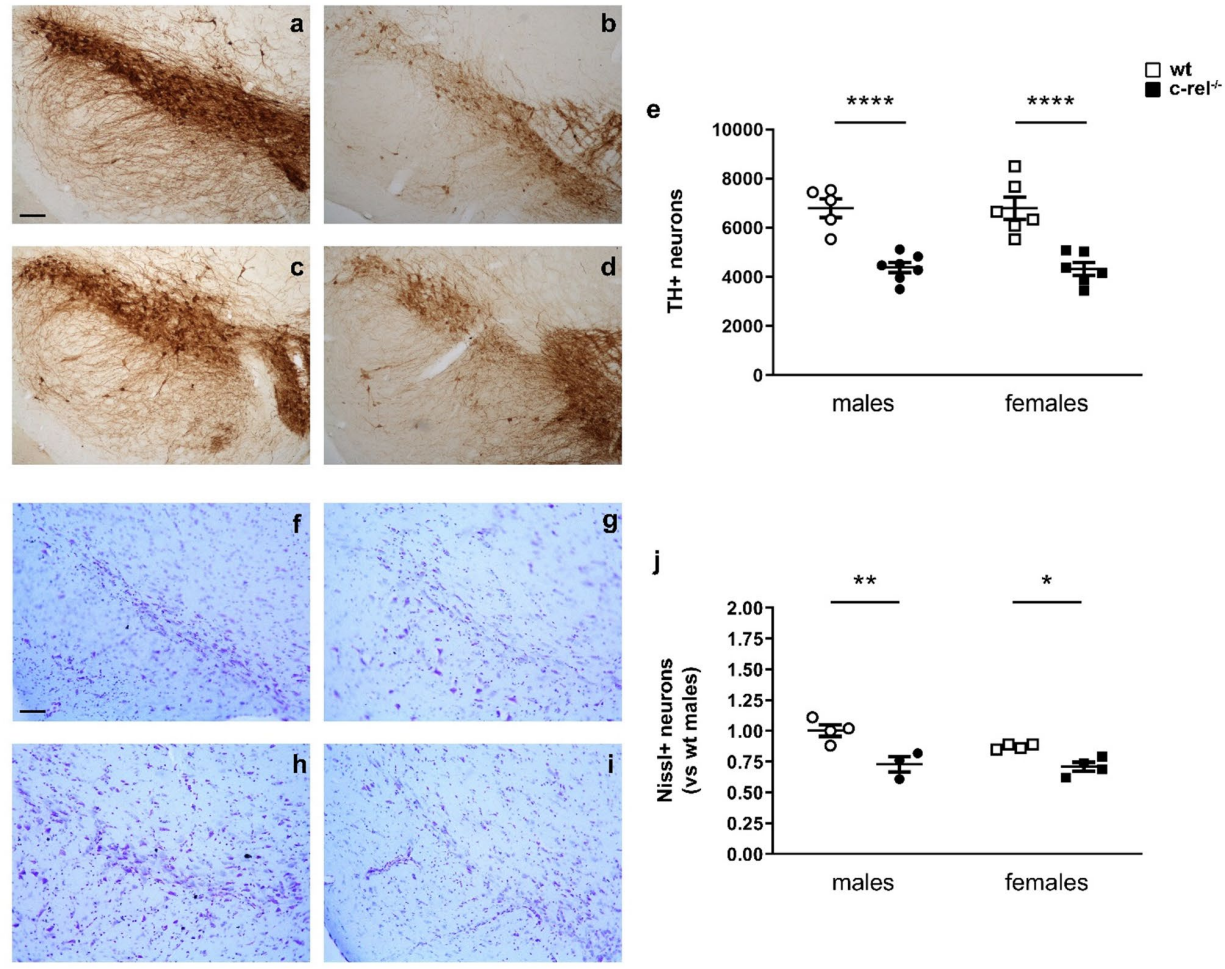
Aged c-rel^-/-^ male and female mice display loss of SNpc dopaminergic neurons. Representative images of TH staining in SN dopaminergic neurons of 18–19-month-old wt males (**a**), c-rel^-/-^ males (**b**), wt females (**c**), c-rel^-/-^ females (**d**). The stereology analysis data of T+ cells are presented in **e**. A significant drop of the number of T+ neurons was observed in both male and female c-rel^-/-^ mice. ****p < 0.0001, two way ANOVA followed by Sidak’s multiple comparison test (wt males: 5 mice; c-rel^-/-^ males: 7; wt females: 6; c-rel^-/-^ females: 6). Representative images of Nissl staining in SN of 18–19-month-old wt males (**f**), c-rel^-/-^ males (**g**), wt females (**h**), c-rel^-/-^ females (**i**). Data from counts of Nissl-stained neurons are presented in **j**. Both male and female c-rel^-/-^ mice showed a significant reduction in Nissl+ neurons. *p < 0.05, **p < 0.01, two way ANOVA followed by Sidak’s multiple comparison test (wt males: 4 mice; c-rel^-/-^ males: 3; wt females: 4; c-rel^-/-^ females: 4). Scale bar in **a** and **f** = 100 μm

To investigate possible sex differences in the nigrostriatal system of c-rel^-/-^ mice, we assessed TH immunoreactivity in SNpc and dorsal striatum of wt and c-rel^-/-^ mice, males and females, at 18–19 months. Both male and female c-rel^-/-^ mice exhibited a marked reduction in the number of TH+ neurons in the SNpc compared to their sex-matched wt counterparts (Fig. 1a-e), highlighting the main effect of genotype on the degeneration of this neuronal population [two-way ANOVA followed by Sidak’s multiple comparison test, Genotype *F*(1, 20) = 55,63, ****p < 0.0001, η_p_^2^ = 0.73. Post-hoc analysis, ****p < 0.0001]. No sexually dimorphic changes were found in both wt and c-rel^-/-^ mice [Sex *F*(1, 20) = 0,007898, *p* > 0.05, η_p_^2^ = 0.00039]. As previously reported [46], the reduction in TH+ neurons observed in male 18–19 month-old c-rel^-/-^ mice was accompanied by a decrease in Nissl-stained neurons in the SNpc. Similar neuronal loss was found in female c-rel^-/-^ mice, indicating that the loss reflects actual dopaminergic neurodegeneration rather than a mere reduction in TH expression (Fig. 1f-j) [Genotype *F*(1, 11) = 28,53, ***p < 0.001, η_p_^2^ = 0.72. Post-hoc analysis, *p < 0.05, **p < 0.01].

**Fig. 2:**
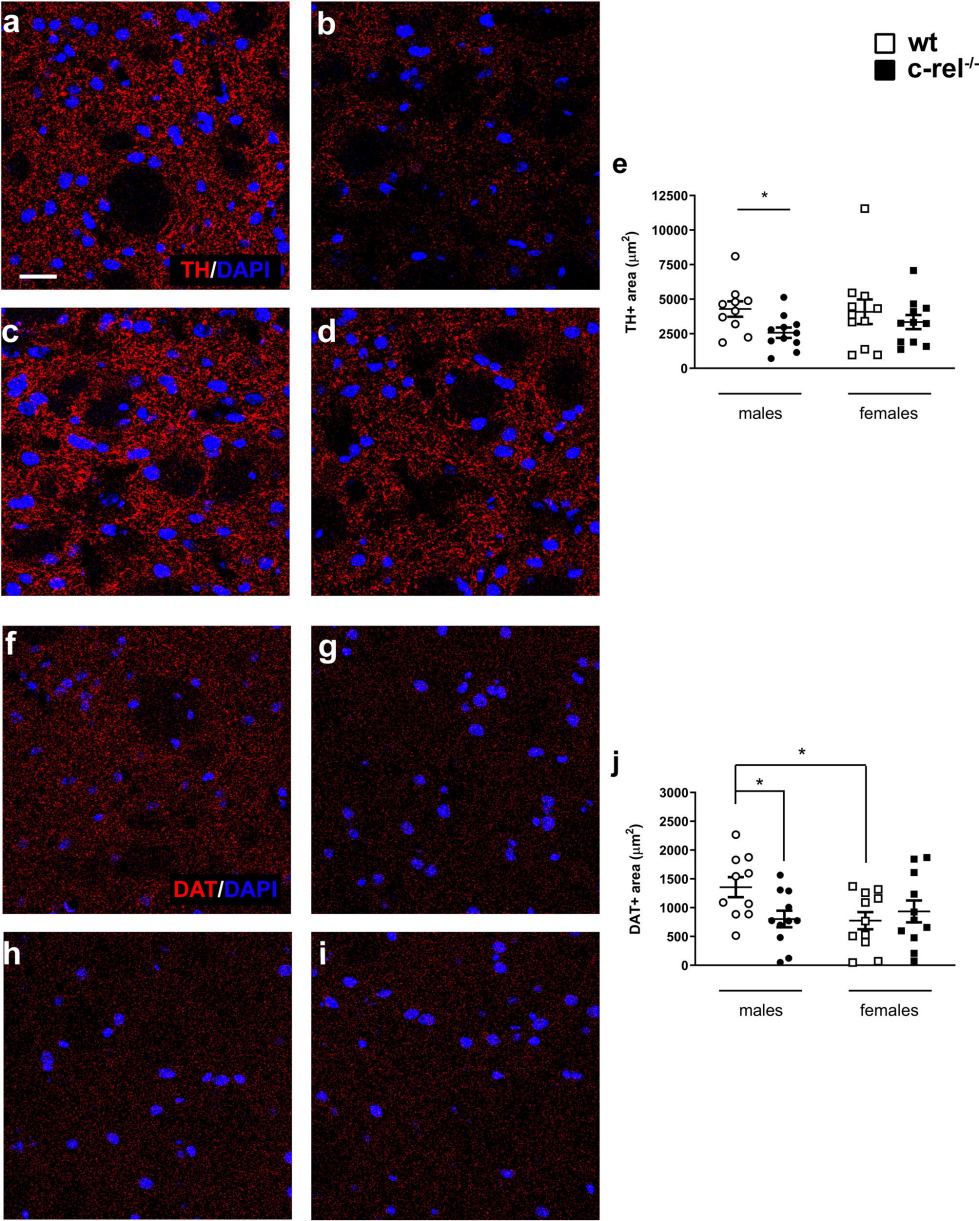
Sexually dimorphic changes in the dorsal striatum of aged c-rel^-/-^ male and female mice. Illustrative pictures of TH immunostaining in caudate putamen of wt males (**a**), c-rel^-/-^ males (**b**), wt females (**c**), c-rel^-/-^ females (**d**). The results from the analysis of TH+ area are shown in **e**. A significant decrease of TH immunoreactivity was observed in c-rel^-/-^ male mice when compared to wt males, but not in c-rel^-/-^ female mice. *p < 0.05, Kruskal-Wallis test followed by Dunn’s multiple comparison test. Representative pictures of DAT immunostaining in caudate putamen of wt males (**f**), c-rel^-/-^ males (**g**), wt females (**h**), c-rel^-/-^ females (**i**). Analysis of DAT+ area is presented in **j**. Wt males displayed significantly higher DAT levels than c-rel^-/-^ males and wt females. *p < 0.05, two way ANOVA followed by Sidak’s multiple comparison test. For both TH and DAT immunostaining the data are presented in µm^2^ as mean ± SEM (*n* = 10–11 animals per group). Scale bar in **a** = 20 μm

At striatal level, we observed a marked reduction of the surface covered by TH+ fibers in c-rel^-/-^ males, but not in females (Fig. 2a-e), (Kruskal-Wallis test followed by Dunn’s multiple comparison test, η^2^ = 0.07. Post-hoc analysis, *p < 0.05). c-rel^-/-^ male mice presented also a significant drop of DAT immunoreactivity (Fig. 2f-j). DAT immunoreactivity was significantly fainter in wt females when compared to wt males [two-way ANOVA followed by Sidak’s multiple comparison test, Genotype * Sex *F*(1, 39) = 4,679, *p < 0.05, η_p_^2^ = 0.11. Post-hoc analysis, *p < 0.05], while the striatal DAT+ areas were comparable between c-rel^-/-^ and wt females.

### Aged c-rel^-/-^ females do not show significant motor deficits

**Fig. 3:**
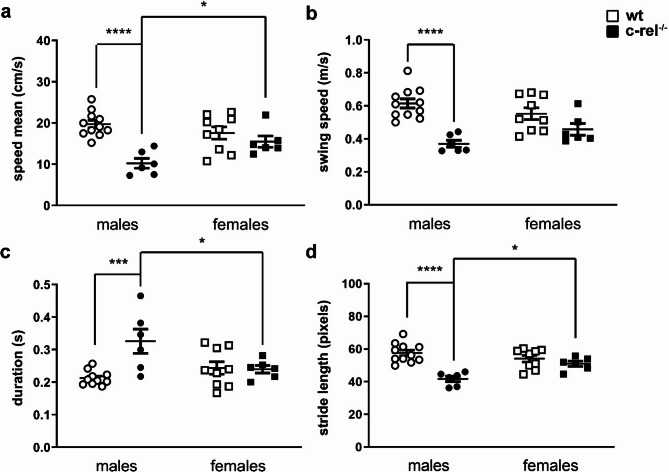
Motor deficits in aged c-rel^-/-^ male and female mice. CatWalk gait analysis was performed in 20–23-month-old wt and c-rel^-/-^ male and female mice (wt males: 11 mice; c-rel^-/-^ males: 6; wt females: 9; c-rel^-/-^ females: 6). c-rel^-/-^ male mice displayed lower run speed (**a**) and swing speed (**b**), higher stand duration (**c**), and lower stride length (**d**) than sex-matched wt mice. Conversely, c-rel^-/-^ females did not significantly differ from wt females in any of the considered gait parameters. *p < 0.05; ***p < 0.001; ****p < 0.0001, two-way ANOVA followed by Sidak’s multiple comparison test

Both males and females, 20–23-month-old wt and c-rel^-/-^ mice were tested for their spontaneous gait behavior with the Catwalk system [46] (Fig. 3 and Additional files 1–4). Run speed, swing speed, stand duration, and stride length were recorded and analyzed. Figure 3a reports data from run speed (cm/seconds) analysis. As also highlighted by the Catwalk videos uploaded as Additional files 1–4, c-rel^-/-^ males were slower to run across the walkway when compared with age-matched wt males [two-way ANOVA followed by Sidak’s multiple comparison test, Genotype *F*(1, 28) = 20,28, ****p < 0.0001, η_p_^2^ = 0.42. Genotype * Sex *F*(1, 28) = 8,272, **p < 0.01, η_p_^2^ = 0.23. Post-hoc analysis, *p < 0.05, ****p < 0.0001]. Figure 3b reports data from evaluation of swing speed (m/seconds). This measures how fast the paw moves during the swing phase, the period in which it is not in contact with the glass plate. c-rel^-/-^ males exhibited a reduced swing speed compared to their wt male siblings [two-way ANOVA followed by Sidak’s multiple comparison test, Genotype *F*(1, 28) = 26,93, ****p < 0.0001, η_p_^2^ = 0.49. Genotype * Sex *F*(1, 28) = 5,231, *p < 0.05, η_p_^2^ = 0.16. Post-hoc analysis, ****p < 0.0001]. Figure 3c shows data from stand duration (seconds) analysis, i.e. the duration of contact of the paws with the glass plate. c-rel^-/-^ males exhibited a longer stand period when compared to wt males [two-way ANOVA followed by Sidak’s multiple comparison test, Genotype *F*(1, 28) = 8,135, **p < 0.01, η_p_^2^ = 0.22. Genotype * Sex *F*(1, 28) = 9,302, **p < 0.01, η_p_^2^ = 0.25. Post-hoc analysis, ***p < 0.001]. Figure 3d reports data from the analysis of stride length (pixels), defined as the distance between successive placements of the same paw. c-rel^-/-^ males showed a shorter forepaw print length in comparison with wt males [two-way ANOVA followed by Sidak’s multiple comparison test, Genotype *F*(1, 28) = 23,97, ****p < 0.0001, η_p_^2^ = 0.46. Genotype * Sex *F*(1, 28) = 10,96, **p < 0.01, η_p_^2^ = 0.28. Post-hoc analysis, ****p < 0.0001]. The abnormal stride length in the c-rel^-/-^ males is clearly visible in the CatWalk videos (Additional files 1–4).

In contrast to c-rel^-/-^ male mice, c-rel^-/-^ females did not significantly differ from corresponding aged-matched wt females in any of the considered gait parameters (two-way ANOVA followed by Sidak’s multiple comparison test).

A significantly better performance was observed in c-rel^-/-^ females compared to c-rel^-/-^ males across all tested gait parameters, except for swing speed (two-way ANOVA followed by Sidak’s multiple comparison test. Post-hoc analysis, *p < 0.05).

### Aged c-rel^-/-^ females do not show significant intestinal dysfunctions

**Fig. 4:**
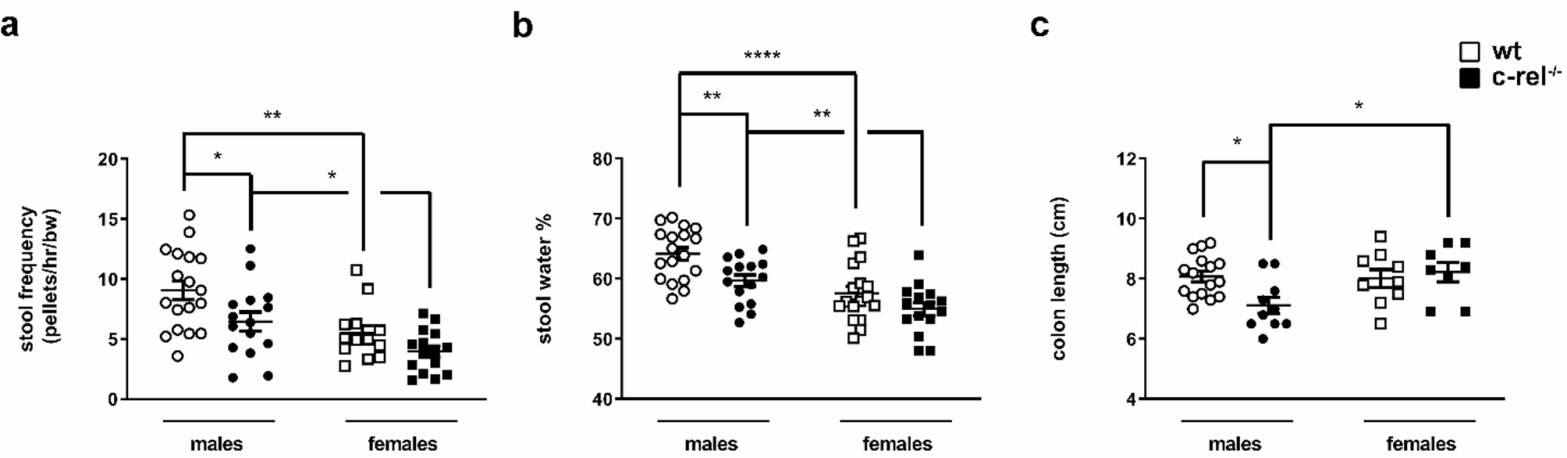
Intestinal dysfunctions in aged c-rel^-/-^ male and female mice. Stool frequency normalized to 30 g of bw (**a**) and stool water content percentage (**b**) of 18–19-month-old wt and c-rel^-/-^ mice, males and females, are displayed (wt males: 18 mice; c-rel^-/-^ males: 15 mice; wt females: 13 mice; c-rel^-/-^ females: 15 mice). Stool frequency and water content percentage are decreased in c-rel^-/-^ male mice, but not in c-rel^-/-^ females. Moreover, both wt and c-rel^-/-^ females showed lower stool frequency and stool water % than corresponding male siblings. (**c**) Colon shortening was investigated in wt and c-rel^-/-^ males and females at 18–20 months. While aged c-rel^-/-^ males displayed a reduction in colon length, c-rel^-/-^ females did not. Wt males: *n* = 16 mice; c-rel^-/-^ males: *n* = 10 mice; wt females: *n* = 9 mice; c-rel^-/-^ females: *n* = 8 mice. *p < 0.05; **p < 0.01; ****p < 0.0001, two-way ANOVA followed by Sidak’s multiple comparisons test. Results were presented as mean ± SEM

One-hour stool collection assay was performed to assess colon motility on both males and females, 18– and 19-month-old wt and c-rel^-/-^ mice [48]. Stool frequency (normalized to bw) and stool water content were significantly reduced in c-rel^-/-^ male mice compared to wt males (Fig. 4a) [stool frequency: two-way ANOVA followed by Sidak’s multiple comparison test, Genotype *F*(1, 57) = 8,555, **p < 0.01 η_p_^2^ = 0.13. Sex *F*(1, 57) = 18,70, ****p < 0.0001, η_p_^2^ = 0.25. Post-hoc analysis, *p < 0.05, **p < 0.01; stool water content: two-way ANOVA followed by Sidak’s multiple comparison test, Genotype *F*(1, 61) = 11,04, **p < 0.01, η_p_^2^ = 0.15. Sex *F*(1, 61) = 27,50, ****p < 0.0001, η_p_^2^ = 0.31. Post-hoc analysis, **p < 0.01, ****p < 0.0001]. Of note, the reduction of colon motility observed in c-rel^-/-^ male mice was not caused by variations in food or water intake (Additional file 5: Fig. 5a, b), since these parameters were not diminished in c-rel^-/-^ mice (two-way ANOVA followed by Sidak’s multiple comparison test).

In contrast, neither stool frequency nor water content differed significantly between c-rel^-/-^ and wt females (two-way ANOVA followed by Sidak’s multiple comparison test).

Next, we analyzed whether aged c-rel^-/-^ female mice would display a milder gut pathology than c-rel^-/-^ males by measuring colon length. Intestinal inflammation, evaluated as decrease of the length of the colon, was observed in c-rel^-/-^ males of 18–20 months, but not in age-matched females [two-way ANOVA followed by Sidak’s multiple comparison test, Sex *F*(1, 39) = 4,106, *p < 0.05, η_p_^2^ = 0.09. Genotype * Sex *F*(1, 39) = 5,175, *p < 0.05, η_p_^2^ = 0.12. Post-hoc analysis, *p < 0.05].

### Aged c-rel^-/-^ females do not show significant olfactory deficits

**Fig. 5:**
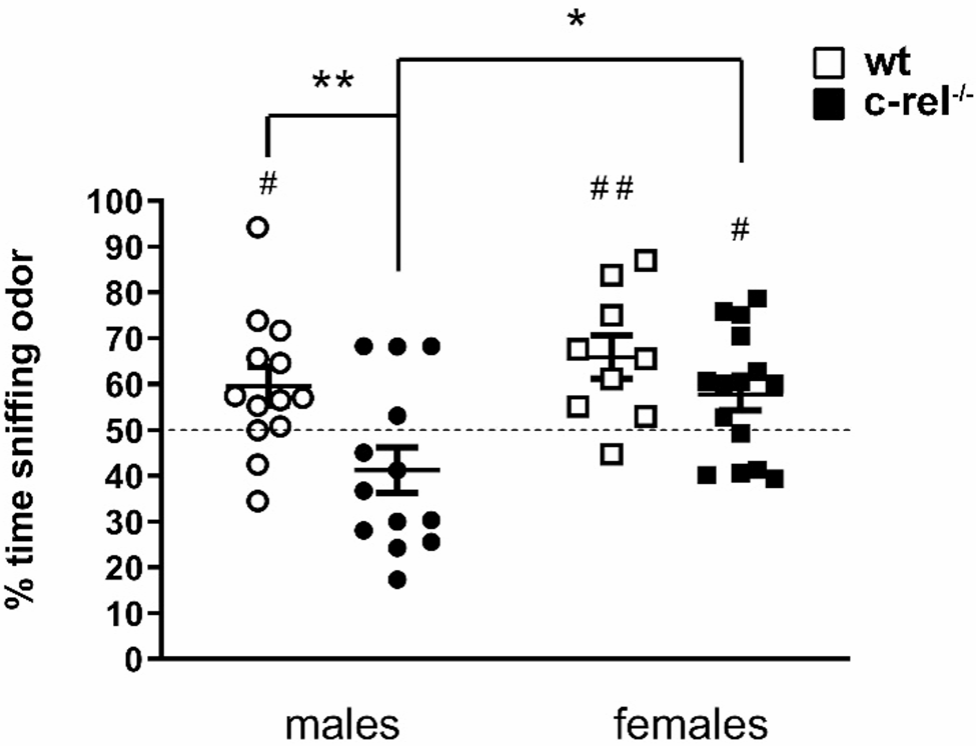
Olfactory deficits in aged c-rel^-/-^ male and female mice. Odor detection test was conducted on 18–19-month-old wt and c-rel^-/-^ males and females (wt males: 13 mice; c-rel^-/-^ males: 13 mice; wt females: 9 mice; c-rel^-/-^ females: 15 mice). The percentage of time sniffing the odor at concentration 1:10^4^ is presented. Aged wt male mice were able to localise the odor, as indicated by the percentage of time sniffing the odor significantly higher than the 50% chance level (#*p* < 0.05, one-sample t-test). On the contrary, age-matched c-rel^-/-^ males could not target the fragrance (*p* > 0.05, one-sample t-test), and exhibited a significant odor detection impairment compared to wt males (*p < 0.05; **p < 0.01, two-way ANOVA followed by Sidak’s multiple comparison test). Both wt and c-rel^-/-^ females recognized the cartridge filled with the vanilla extract (# #*p* < 0.01 and #*p* < 0.05, respectively, one-sample t-test)

Olfactory deficits have been analyzed by testing 18–19-month-old wt and c-rel^-/-^ mice, males and females, for their olfactory threshold with the odor detection test. The test evaluates the mice ability to perceive odors by measuring the time spent sniffing two cartridges: one containing water and the other containing vanilla extract. Animals with intact olfactory function naturally dedicate more than 50% of their time (chance level) smelling the cartridge filled with vanilla extract, whereas those with olfactory deficits display no preference between the two cartridges (percentage of time sniffing the odor similar to the level of chance) [48]. Neither aged wt nor c-rel^-/-^ mice of both sexes were capable of detecting the lowest odor concentrations [Additional file 5: Fig. 5c (dilution 1:10^8^) and Additional file 5: Fig.  5d (dilution 1:10^6^), one-sample t-test vs. chance level, *p* > 0.05]. At the highest vanilla concentration [Fig. 5 (dilution 1:10^4^)] wt males could locate the odor (one-sample t-test vs. chance level, *p < 0.05, *d* = 0.63), while c-rel^-/-^ males failed (one-sample t-test vs. chance level, *p* > 0.05, *d* = -0.48). Furthermore, c-rel^-/-^ males spent a significantly lower percentage of time sniffing the odor compared to both wt males and c-rel^-/-^ females [two-way ANOVA followed by Sidak’s multiple comparison test, Genotype *F*(1, 46) = 8,890, **p < 0.01, η_p_^2^ = 0.16. Sex *F*(1, 46) = 6,704, *p < 0.05, η_p_^2^ = 0.13. Post-hoc analysis, *p < 0.05, **p < 0.01]. On the other hand, both wt and c-rel^-/-^ females successfully identified the odorous cartridge (one-sample t-test vs. chance level, **p < 0.01 and *p < 0.05, *d* = 1.12 and *d* = 0.58 respectively). The total sniffing time scored at the odor dilution 1:10^4^ did not change between wt and c-rel^-/-^ mice of both sexes, suggesting that the bad performance of c-rel^-/-^ males in locating the odor was not caused by an altered exploratory behavior (Additional file 5: Fig. e. two-way ANOVA followed by Sidak’s multiple comparison test).

### Aged c-rel^-/-^ females exhibit milder neuropsychiatric deficits compared to their c-rel^-/-^ male counterparts

**Fig. 6:**
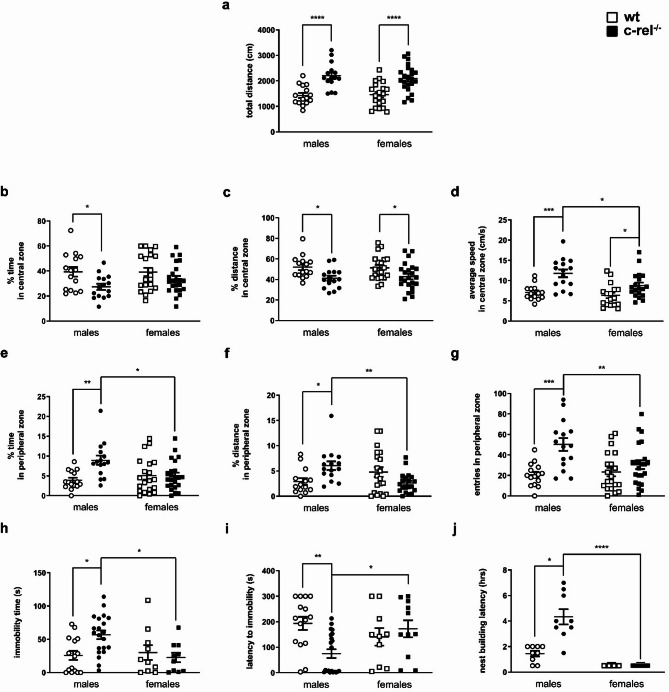
Neuropsychiatric symptoms in aged c-rel^-/-^ male and female mice. Anxiety-like behavior was assessed by the OF test (**a**-**g**) in 18–19-month-old wt and c-rel^-/-^ mice, males and females (wt males: 15 mice; c-rel^-/-^ males: 15 mice; wt females: 20 mice; c-rel^-/-^ females: 23 mice). The total distance covered (**a**), the time spent, the distance covered, and the average speed scored in the center of the apparatus (**b-d**, respectively), the time spent, the distance covered, and the number of entries calculated in the peripheral area (**e-g**, respectively) are displayed. c-rel^-/-^ male and female mice shared a similar behavior in comparison with sex-matched controls, covering a higher total distance (**a**, males and females *p* < 0.0001), and travelling a lower distance (**c**, males and females *p* < 0.05) but at a higher speed (**d**, males *p* < 0.001, females *p* < 0.05) in the central area. Conversely, while c-rel^-/-^ males spent less time in the central area (**b**, *p* < 0.05), and spent more time, covered a higher distance and moved into the peripheral area more frequently (**e**: *p* < 0.05, **f**: *p* < 0.05, **g**: *p* < 0.001, respectively) in comparison to wt males, c-rel^-/-^ females did not differ significantly from wt females in these parameters (**b**, **e-g**: *p* > 0.05). Depressive-like behavior was measured with the FST in wt and c-rel^-/-^ mice aged 18–19 months, males and females (wt males: 15 mice; c-rel^-/-^ males: 21 mice; wt females: 10 mice; c-rel^-/-^ females: 11 mice). The latency to immobility (**h**) and the immobility time (**i**) are shown. A higher immobility time and a shorter latency to immobility were observed in c-rel^-/-^ male mice compared with sex-matched wt (**h**: *p* < 0.05, **i**: *p* < 0.01, respectively), but not in c-rel^-/-^ females vs. wt females (**h**, **i**: *p* > 0.05). Apathetic behavior was measured as latency to build the nest in the nest building test (**j**) in 18–19-month-old wt and c-rel^-/-^ mice, males and females (wt males: 9 cages; c-rel^-/-^ males: 9 cages; wt females: 7 cages; c-rel^-/-^ females: 8 cages). c-rel^-/-^ male mice took more time to build the nest in comparison to sex-matched wt (**j**: *p* < 0.05), while c-rel^-/-^ females did not (**j**: *p* > 0.05). c-rel^-/-^ females were faster than corresponding males in building the nest (**j**: *p* < 0.0001, respectively). *p < 0.05; **p < 0.01; ***p < 0.001, ****p < 0.0001. Two-way ANOVA followed by Sidak’s multiple comparison test in (**a-d**, **f**); Kruskal-Wallis test followed by Dunn’s multiple comparison test in (**e**, **g-j**)

Anxiety-like behavior was assessed by the OF test [49] in 18–19-month-old wt and c-rel^-/-^ mice, males and females. c-rel^-/-^ male mice positioned in the center of a black plastic OF arena and analyzed for 5 min displayed an anxious behavior (Fig. 6a-g). In comparison with sex-matched wt mice, they covered a higher total distance [two-way ANOVA followed by Sidak’s multiple comparison test, Genotype *F*(1, 69) = 37,99, ****p < 0.0001, η_p_^2^ = 0.35. Post-hoc analysis, ****p < 0.0001], spent less time and traveled a shorter distance but faster in the central zone [time in central zone: two-way ANOVA followed by Sidak’s multiple comparison test, Genotype *F*(1, 69) = 8,049, **p < 0.01, η_p_^2^ = 0.10. Post-hoc analysis, *p < 0.05; distance in central zone: two-way ANOVA followed by Sidak’s multiple comparison test, Genotype *F*(1, 69) = 12,20, ***p < 0.001, η_p_^2^ = 0.15. Post-hoc analysis, *p < 0.05; average speed in central zone: two-way ANOVA followed by Sidak’s multiple comparison test, Genotype *F*(1, 61) = 22,81, ****p < 0.0001, η_p_^2^ = 0.27. Sex *F*(1, 61) = 6,191, *p < 0.05, η_p_^2^ = 0.09. Post-hoc analysis, *p < 0.05, ***p < 0.001]. c-rel^-/-^ males spent significantly more time, traveled a greater distance, and entered more frequently in the peripheral zone [time in peripheral zone: Kruskal-Wallis test followed by Dunn’s multiple comparison test, η^2^ = 0.13. Post-hoc analysis, *p < 0.05, **p < 0.01; distance in peripheral zone: Kruskal-Wallis test followed by Dunn’s multiple comparison test, η^2^ = 0.13. Post-hoc analysis, *p < 0.05, **p < 0.01; entries in peripheral zone: two-way ANOVA followed by Sidak’s multiple comparison test, *F*(1, 69) = 16,08, ***p < 0.001, η_p_^2^ = 0.19. Genotype * Sex *F*(1, 69) = 6,005, *p < 0.05, η_p_^2^ = 0.08. Post-hoc analysis, **p < 0.01, ***p < 0.001].

Similarly to c-rel^-/-^ males, c-rel^-/-^ females were significantly more active, and covered a shorter distance but moved faster in the central area. Though, c-rel^-/-^ females did not significantly differ from wt females in the time spent in the central area and in the locomotion parameters related to the peripheral area (Kruskal-Wallis test followed by Dunn’s multiple comparison test; two-way ANOVA followed by Sidak’s multiple comparison test).

Depressive-like behavior was investigated by the FST in wt and c-rel^-/-^ mice, males and females, of 18–19 months (Fig. 6h, i) [49]. A higher immobility time and a shorter latency to immobility, two indicators of depressive-like behavior, were found in c-rel^-/-^ male mice compared with sex-matched wt, but not in c-rel^-/-^ females (immobility time: Kruskal-Wallis test followed by Dunn’s multiple comparison test, η^2^ = 0.19. Post-hoc analysis, *p < 0.05; latency to immobility: Kruskal-Wallis test followed by Dunn’s multiple comparison test, η^2^ = 0.20. Post-hoc analysis, *p < 0.05, **p < 0.01). The number of wheel rotations and turning time together with maximum and average rpm did not differ among wt and c-rel^-/-^ mice in either sex (Additional file 5: Fig. 5g-i, two-way ANOVA followed by Sidak’s multiple comparison test). Also the bw, a parameter influencing rodents’ performance in the FST [59], did not vary among wt and c-rel^-/-^ males and females (Additional file 5: Fig. 5f, two-way ANOVA followed by Sidak’s multiple comparison test).

Apathetic behavior was analyzed by the nest building test in 18–19-month-old wt and c-rel^-/-^ mice, males and females (Fig. 6j) [49]. While c-rel^-/-^ males needed more time to nibble the cellulose bag and prepare a nest in comparison with sex-matched wt mice, c-rel^-/-^ females did not show any difference from wt females (Kruskal-Wallis test followed by Dunn’s multiple comparison test, η^2^ = 0.83. Post-hoc analysis, *p < 0.05). Moreover, c-rel^-/-^ females were significantly faster than corresponding male siblings in building the nest (Kruskal-Wallis test followed by Dunn’s multiple comparison test, η^2^ = 0.83. Post-hoc analysis, ****p < 0.0001).

## Discussion

This study highlights sexual dimorphism in the c-rel^-/-^ mouse model of PD, as aged female mice exhibit preserved nigrostriatal dopaminergic fibers and lack of significant motor and prodromal deficits. In contrast to aged c-rel^-/-^ male mice displaying a significant reduction of TH+ neurons in the SN and TH+ fiber density in the striatum [46, 48], aged female c-rel^-/-^ mice showed a loss of TH+ neuronal population in the SN with a conserved density of striatal dopaminergic fibers. The preservation of dopaminergic fiber density may point to the possibility of a trophic process that could support striatal dopaminergic fibers in females over the course of their lifespan.

Striatal DAT levels were also reduced in male c-rel^-/-^ mice compared their wt littermates, in line with previous findings [46]. In contrast, no changes in DAT immunoreactivity were observed between wt and c-rel^-/-^ females. Interestingly, wt females exhibited significantly lower DAT density compared to wt males. Given the comparable density of dopaminergic fiber between sexes, it may be speculated that reduced DAT expression can enhance dopaminergic transmission efficiency in aged wt females. The preservation of striatal dopaminergic fiber and DAT expression in c-rel^-/-^ females, despite the loss of SN dopaminergic neurons, likely explains the lack of a parkinsonian behavioral phenotype in these mice. A similar difference between male and females was also found in human subjects. Imaging studies demonstrated a more rapid age-related decline of striatal DAT activity in women than men in both healthy subjects and PD patients [60, 61].

When analyzed from the behavioral point of view, the catwalk gait parameters confirmed gait-related deficits in aged c-rel^-/-^ male mice [46]. Similarly to MPTP-treated and 6-OHDA-treated rodent models of PD, male c-rel^-/-^ mice were slower in traveling along the catwalk [62–65]. In accordance with previous studies on other PD rodent models [62–67], c-rel^-/-^ males exhibited a reduced swing speed, a feature associated with bradykinesia [68]. In addition, male c-rel^-/-^ mice displayed an increase on stand duration compared to wt mice, as reported in other PD animal models [63, 65, 67]. This parameter mimics the freezing of gait that can be caused by an increase in muscle rigidity [69]. Finally, stride length during walking was reduced in c-rel^-/-^ males. Decreased stride length during walking is a feature often seen in PD patients [70], and was also found in toxin-induced models of PD [64, 65, 67, 71]. All these gait parameters were not significantly altered in c-rel^-/-^ females, suggesting a sex-dependent differential motor phenotype in this PD mouse model, reminiscent of the milder motor symptoms observed in women affected by PD [10, 12].

The lack of significant motor deficits in aged c-rel^-/-^ female mice is consistent with the different degenerative process occurring in the two sexes. Females c-rel^-/-^ mice displayed a milder dopaminergic neurodegeneration of the nigrostriatal tract compared with males. Aged c-rel^-/-^ female mice did not differ from wt littermates in terms of striatal dopaminergic fiber integrity and DAT levels. Notably, wt female mice exhibited lower DAT levels compared to wt males, which however, did not impact on motor behavior. Indications for a sexual dimorphism in the nigrostriatal pathway in PD come from both preclinical and clinical studies. Similarly to the c-rel^-/-^ model, in mice expressing the human A53T variant of α-synuclein (A53T mice), females developed milder motor impairment than males, and displayed loss of dopaminergic neurons in SN without degeneration of dopaminergic fibers in the striatum [72]. In healthy rats, DAT density and striatal dopamine levels were higher in females than in males [73]. In toxin-based rodent models of PD, the loss of nigral dopaminergic neurons and the reduction of striatal dopamine levels is more marked in males, while females are more resistant to low doses of toxins and developed a lower PD-like pathology [74–76]. In a similar fashion, in the MitoPark mouse model of PD, females presented a delayed presynaptic dopamine dysfunctions in the nigrostriatal pathway and delayed motor symptoms than males [77].

The more benign PD phenotype with delayed disease onset in women is associated with preserved global gray matter volumes. Magnetic resonance imaging in *de novo* male and female PD patients revealed that women showed significant larger volumes in several subcortical structures, including the caudate and putamen, relative to male patients [78].

Differential mitochondrial functions and the role of estrogens are among the key factors contributing to the sexual dimorphism observed at the nigrostriatal level [7]. Mitochondrial function and resistance to oxidative stress exhibit sexual dimorphism across various brain regions, including the striatum [79]. For instance, female mice demonstrate lower mitochondrial ROS production in the striatum compared to males, attributed to higher antioxidant defenses [80–82]. Supporting this, mesencephalic neurons from male mice exposed to 6-OHDA show increased ROS levels, along with reduced ATP and mitochondrial protein levels, compared to neurons from females [83].

Estrogens are proposed to play a neuroprotective role through their neurotrophic, antioxidant and anti-inflammatory effects, as well as their regulatory influence on the nigrostriatal dopaminergic system [7, 84]. Different types of estrogen receptors (ERs) are present in brain regions including SN and striatum. Interestingly, ERs are not expressed in dopaminergic neurons themselves but are localized in the surrounding cellular microenvironment, including interneurons, glia and microglia, which interact with nigrostriatal TH+ neurons [7]. Estrogens can exert a neurotrophic activity by enhancing brain-derived neurotrophic factor (BDNF) expression [85] and mitigate oxidative stress by upregulating glial cell–derived neurotrophic factor (GDNF) and B-cell lymphoma 2 (Bcl-2) [86]. Although sexual dimorphism in neuroinflammation has been scarcely investigated in PD patients and preclinical models, estrogens may also be beneficial by modulating microglia activity [87]. Finally, estrogens have also been reported to positively affect DAT levels and the dopamine synthesis [60].

Behavioral abnormalities in aged c-rel^-/-^ female mice were evaluated using non-motor tasks. Assessment of stool frequency and stool water content in aged wt and c-rel^-/-^ mice of both sexes confirmed a reduced colon motility in c-rel^-/-^ males [48]. Conversely, c-rel^-/-^ females did not show significant constipation. Interestingly, both wt and c-rel^-/-^ females showed reduced stool frequency and stool water content compared to age-matched males. This finding aligns with the fact that constipation is commonly observed in postmenopausal women [88].

In the odor detection test, consistent with our prior results [48], aged c-rel^-/-^ males showed reduced ability to locate the odor compared to wt males, while both aged wt and c-rel^-/-^ females successfully detected the scent. Potential confounding factors may affect olfactory-driven behavior in this task. Notably, sex-specific differences in odor preferences influenced by mating and breeding behaviors, as well as modulation of olfactory sensitivity by sex hormones, have been documented in rodents [89]. Although we cannot rule out sex-specific differences in the detection of certain compounds, these results suggest preserved olfactory function in aged c-rel^-/-^ females. Furthermore, they are consistent with studies indicating more pronounced olfactory deficits in male than female PD patients [19, 23, 90].

The results obtained with the OF test, FST and nest building task confirmed that aged c-rel^-/-^ male mice exhibit neuropsychiatric symptoms, including anxiety-, depressive- and apathetic-like behaviors [49]. Aged c-rel^-/-^ females exhibited an anxious behavior in some parameters scored in the OF test. Notably, the absence of differences between males and females in total distance traveled during the OF test is consistent with previous studies in other PD mouse models [91]. In contrast, aged c-rel^-/-^ female mice did not display depressive-like behavior when subjected to the FST. Also, aged wt and c-rel^-/-^ females performed similarly in the nest building test. Of note, consistent with prior findings [92], aged wt females were faster than wt males in building a nest. While we cannot exclude the presence of intrinsic sex differences that may have influenced the performances of male and female mice in the different behavioral tests, these results would suggest that aged female c-rel^-/-^ mice display a milder PD-like phenotype, in comparison with age-matched male mice of the same strain.

Several mechanisms may contribute to the sexually dimorphic non-motor symptomatology in c-rel^-/-^ mice. For example, our preliminary observation of absent colon shortening in c-rel^-/-^ females suggest milder intestinal inflammation. Gastrointestinal inflammation has been observed in PD patients, correlating with disease severity [93]. At the olfactory level, neurodegeneration and microgliosis have been reported in the OBs of male PD patients, but not female, suggesting that these factors may contribute to the sex differences in hyposmia [94]. Additionally, a recent proteomic study revealed sex-dependent changes in Sirtuin signaling in the olfactory tract of PD patients [95].

A limitation of this study is that it provides a snap of a specific point in the progression of c-rel^-/-^ mice pathology, namely the old age when motor deficits begin to be detectable. Several imaging studies have shown that women have higher striatal DAT activity than men during young-to-middle age, a feature likely influenced by estrogens [57, 96–98]. Estrogen levels reach their highest during the reproductive lifespan, the period between menarche and menopause, allowing women to benefit the most from estrogen exposure [99]. Observational studies have reported a direct correlation between duration of reproductive lifespan in female PD patients and the onset, progression and severity of motor symptoms, with a shortened reproductive lifespan being associated with an early, faster and more severe disease disability [99–101]. Thus, reproductive factors in females might aid in mitigating PD progression. In light of these considerations, we speculate that the milder symptomatology observed in aged c-rel^-/-^ females may be due to estrogens exposure during their reproductive lifespan. Future studies investigating the symptoms and pathology of c-rel^-/-^ females at younger ages will be necessary to test these hypotheses.

Finally, several studies pointed out a sexual dimorphism in NF-κB activity [102–105]. In particular, recent trascriptomic analyses have also indicated sex-dependent differences for NF-κB members, including RelA, NF-κB1 and RelB, in PD [106–109]. Hence, it will be crucial to determine whether sexually dimorphic activity of NF-κB subunits may be involved in the milder pathology and symptomatology observed in the females of this mouse model of PD.

### Perspectives and significance

In summary, when compared with aged c-rel^-/-^ male mice, age-matched c-rel^-/-^ females displayed a milder pathology, characterized by the loss of SNpc dopaminergic neurons without a significant loss of striatal dopaminergic fibers and terminals. In addition, c-rel^-/-^ females suffer from milder motor symptoms, constipation, olfactory dysfunctions and neuropsychiatric deficits. The sexually dimorphic phenotype found in c-rel^-/-^ mouse model of PD aligns with sex differences observed in PD patients, supporting the validity of these animals as a preclinical model of PD.

## Conclusions

In conclusion, the results showing lower vulnerability to striatal dopaminergic degeneration and less severe symptoms in c-rel^-/-^ female mice indicate the suitability of this mouse model to study sex differences in PD. Clarifying the role of sex in PD will be essential to develop sex-specific diagnostic, preventive and therapeutic strategies for this disorder.

## Supplementary Information

Supplementary Material 1

Supplementary Material 2

Supplementary Material 3

Supplementary Material 4

Supplementary Material 5

## Data Availability

All data generated and/or analysed during the current study are available from the corresponding author on reasonable request.
