# Supplementary figures and images for: Sex-related differences in phenotype and nigro-striatal degeneration of c-rel-/- mouse model of Parkinson’s disease
Source: Biol Sex Differ. 2025 Oct 10;16:73. doi: 10.1186/s13293-025-00761-0 (PMC12512402; doi:10.1186/s13293-025-00761-0)

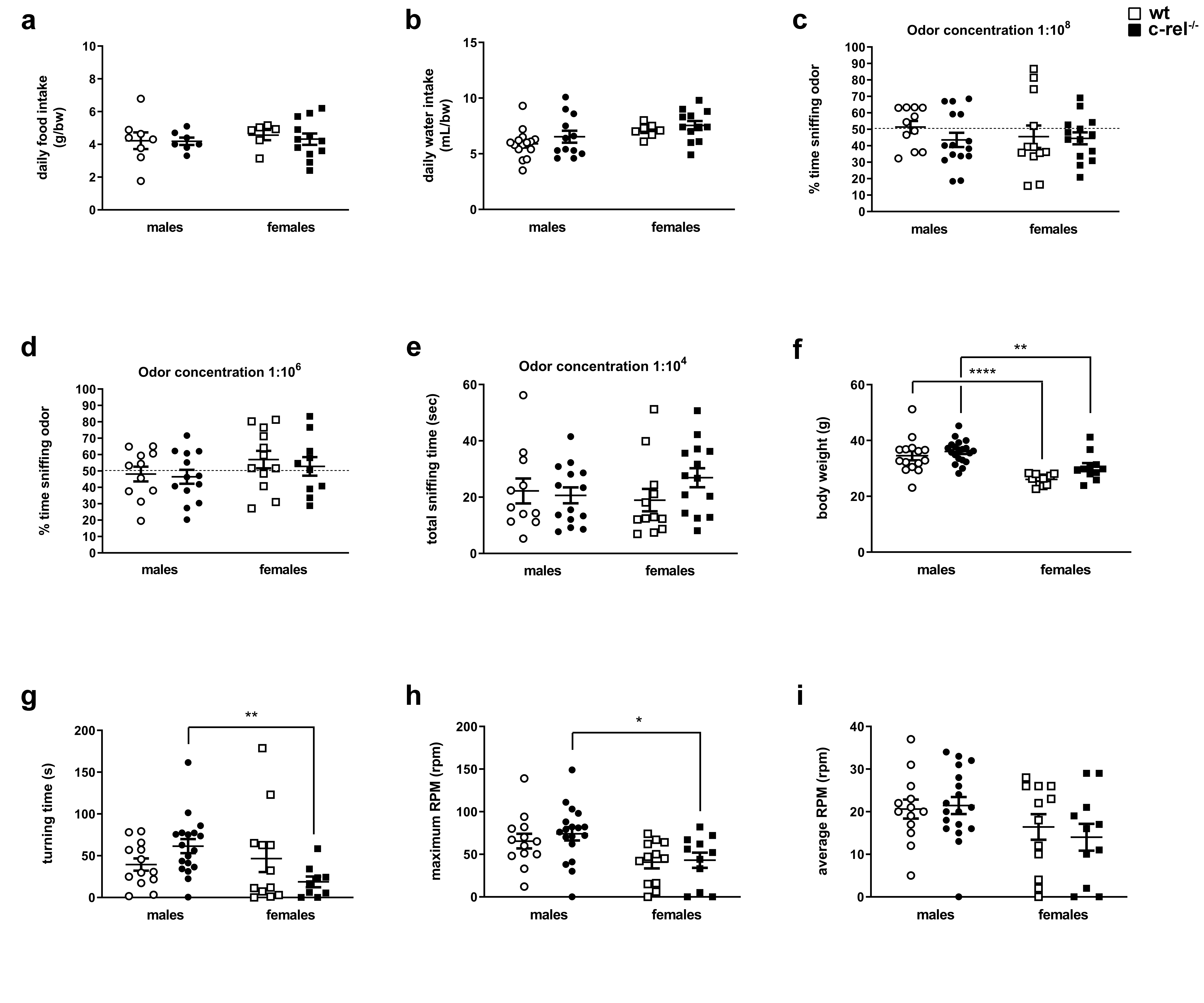

Supplement: Supplementary file 5 — Supplementary Material 5 [file 13293_2025_761_MOESM5_ESM.tif]
